# A new blue zone? exceptional late-life survival in Bolotana, Sardinia

**DOI:** 10.3389/fragi.2026.1951042

**Published:** 2026-10-05

**Authors:** Giovanni Mario Pes, Steven N. Austad, Dan Buettner, Salvatore Pedde, Vittorio Romerio, Hiram Beltrán-Sánchez, S. Jay Olshansky

**Affiliations:** 1 Department of Medicine, Surgery and Pharmacy, University of Sassari, Sassari, Italy; 2 Department of Biology, University of Alabama at Birmingham, Birmingham, AL, United States; 3 Blue Zones, Edina, MN, United States; 4 Orminas Cultural Association, Bolotana, Sardinia, Italy; 5 Scuola di Archivistica, Paleografia e Diplomatica dell'Archivio di Stato di Cagliari, Alghero, Sardinia, Italy; 6 Department of Community Health Sciences and California Center for Population Research, University of California, Los Angeles (UCLA), Los Angeles, CA, United States; 7 University of Illinois, Chicago, IL, United States

**Keywords:** blue zones, exceptional longevity, human longevity, late-life survival, life expectancy, Sardinia, small populations

## Abstract

**Background:**

Exceptional longevity in Sardinia has historically been associated with the original Sardinian Blue Zone. We investigated whether Bolotana, a small mountain municipality outside that area, exhibits a distinct late-life survival profile.

**Methods:**

Using validated civil and parish records, we constructed sex-specific partial period life tables for 2021–2025. Late-life survival was defined as survival from age 70 onward and assessed through remaining life expectancy at age 70 (e_70_) and conditional survival from age 70 to 100 [S_(70→100)_]. Estimates were compared descriptively with Nuoro Province, Sardinia, and Italy, the original Sardinian Blue Zone, and published international benchmark. Statistical uncertainty was evaluated using parametric Poisson bootstrap. Sensitivity analyses included 2020–2025 and a period-matched 2015–2019 analysis.

**Results:**

In 2021–2025, e_70_ was 16.02 years in men (95% CI, 14.55–17.79) and 20.85 years in women (19.42–22.43); S_(70–100)_ was 4.92% (1.58–12.76) and 13.83% (7.33–23.75), respectively. Female estimates _(70–100)_ exceeded contemporary values for Nuoro Province, Sardinia, and Italy. Among international benchmarks, Bolotana women ranked second only to Hong Kong for both indicators, and S_(70→100)_ was more than three times that of U.S. women. Inclusion of 2020 to address the potential effects of COVID-19 produced modest reductions, whereas the period-matched 2015–2019 analysis yielded a less pronounced late-life survival profile, particularly for survival to age 100.

**Conclusion:**

Bolotana exhibited a distinctive, predominantly female pattern of exceptional late-life survival and may be considered a candidate longevity blue zone. However, the magnitude of this advantage varied by calendar period. Longer observation and continued demographic validation, are required before Bolotana can be considered an established longevity blue zone.

## 1 Introduction

Human period life expectancy at birth has increased markedly over the past two centuries, although the magnitude and pace of improvement have varied across and within populations. Some geographically circumscribed populations exhibit exceptionally favorable survival relative to their national or regional contexts. These populations, commonly referred to as blue zones after the blue markings used in the original mapping of Sardinian longevity (Poulain et al., 2004), provide valuable settings to investigate the demographic, social, behavioral, and environmental correlates of exceptional survival.

The original blue zones emerged without planned population-level interventions and were identified independently based on unusually favorable longevity indicators. The historically documented populations in Okinawa (Japan), Sardinia (Italy), and Nicoya (Costa Rica) have subsequently been reassessed using standardized demographic criteria (Olshansky et al., 2026). This recent evidence suggests that the longevity advantage of some of these populations has weakened over time, particularly in Nicoya and Okinawa (Hokama and Binns, 2008; Rosero-Bixby, 2023). Possible explanations include (i) catch-up among national and other long-lived populations, (ii) a general slowdown in late-life mortality improvement consistent with increasing dominance of aging-related causes at advanced ages, and (iii) cohort replacement that erodes historically favorable early-life, midlife, and exposures that characterized early-20th-century cohorts in these regions. Against this background, we examined late-life survival in Bolotana, Sardinia, a village located approximately 110 km from the originally identified Sardinian blue zone. In the present study, late-life survival refers to survival from age 70 onward and is characterized by two complementary period life-table indicators: remaining life expectancy at age 70 (e_70_) and conditional survival from age 70 to exact age 100 [S_(70→100)_].

Bolotana is a mountain community in the Marghine area of central Sardinia, outside the historically identified Sardinian Blue Zone (Figure 1). Its long-standing demographic continuity and unusually complete civil and parish records make it particularly suitable for the study of exceptional longevity. The objective of the present study was to determine whether Bolotana exhibits a demographic profile compatible with a candidate longevity blue zone. To this end, we evaluated sex-specific remaining life expectancy at age 70 and conditional survival from age 70 to age 100 in Bolotana during 2021–2025. These indicators were compared with contemporary values for Nuoro Province, Sardinia, and Italy. To provide a more stringent benchmark of established exceptional longevity, Bolotana was also compared with published pooled estimates for the 14 municipalities constituting the original Sardinian Blue Zone during 2015–2019 (Olshansky et al., 2026). The robustness of the findings was evaluated using alternative assumptions for life-table construction and statistical uncertainty.

**FIGURE 1:**
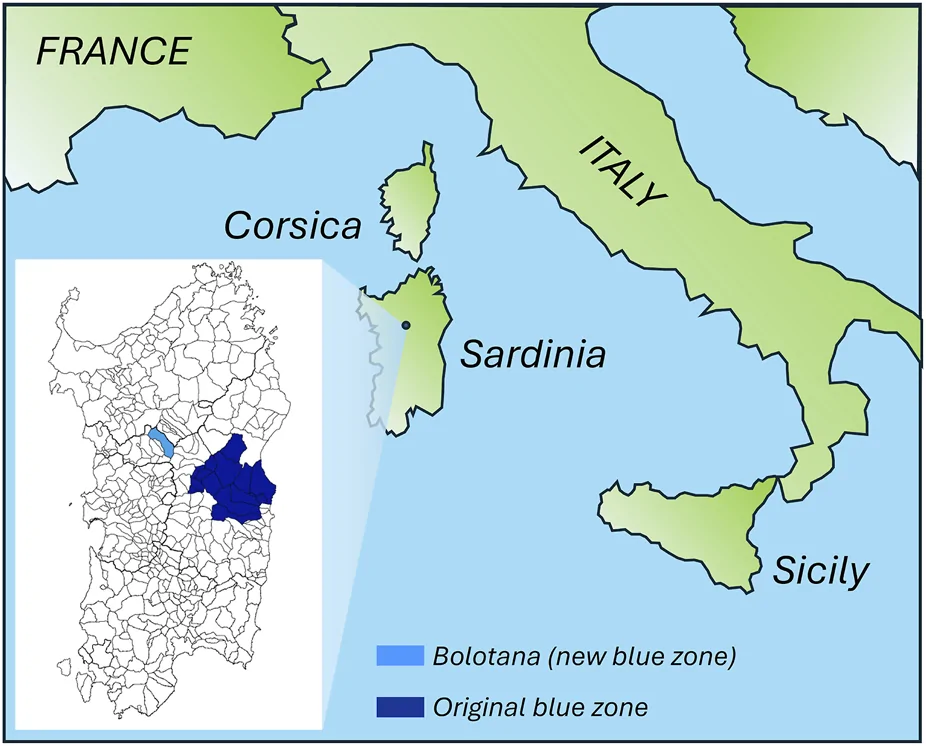
Geographic location of Bolotana, and the original Sardinian Blue Zone. The main map situates Sardinia within the western Mediterranean in relation to Corsica, mainland Italy, Sicily, and southern France. The inset shows the municipalities of Sardinia, with Bolotana highlighted in pale blue and the 14 municipalities constituting the original Sardinian Blue Zone highlighted in deep blue.

## 2 Methods

### 2.1 Study population and age validation

Bolotana is a small municipality in central Sardinia characterized by substantial residential continuity and extensive historical demographic documentation. The primary period analysis included individuals registered as residents of Bolotana aged 70 years or older and alive at some point during 2021–2025. Demographic information was obtained from the municipal civil registry, and included sex, dates and places of birth and death, residence, and migration status. Historical parish registers provided an independent source for cross-validation of births and baptisms, deaths, as well as burials. Civil birth records were linked to subsequent civil and, when available, parish records using concordance of name, sex, dates, parentage, family relationships, and other identifying information. Genealogical timelines were additionally reconstructed when necessary to exclude linkage to siblings or homonymous relatives. Individuals who were not officially resident in Bolotana were not included in the resident mortality numerator.

Civil birth documentation was available for all identified centenarians, and 97% of recorded centenarian deaths were independently cross-checked against parish archival records Figure 2. On the basis of these cross-checks, the ages of all identified centenarians living or deceased in Bolotana were validated (Austad and Pes, 2025). No centenarian case was excluded because of insufficient age documentation. Approximately 3% of individuals born in Bolotana could not be followed to a documented place and date of death, principally because of out-migration; this issue is relevant primarily to the cohort-based analysis described below.

**FIGURE 2:**
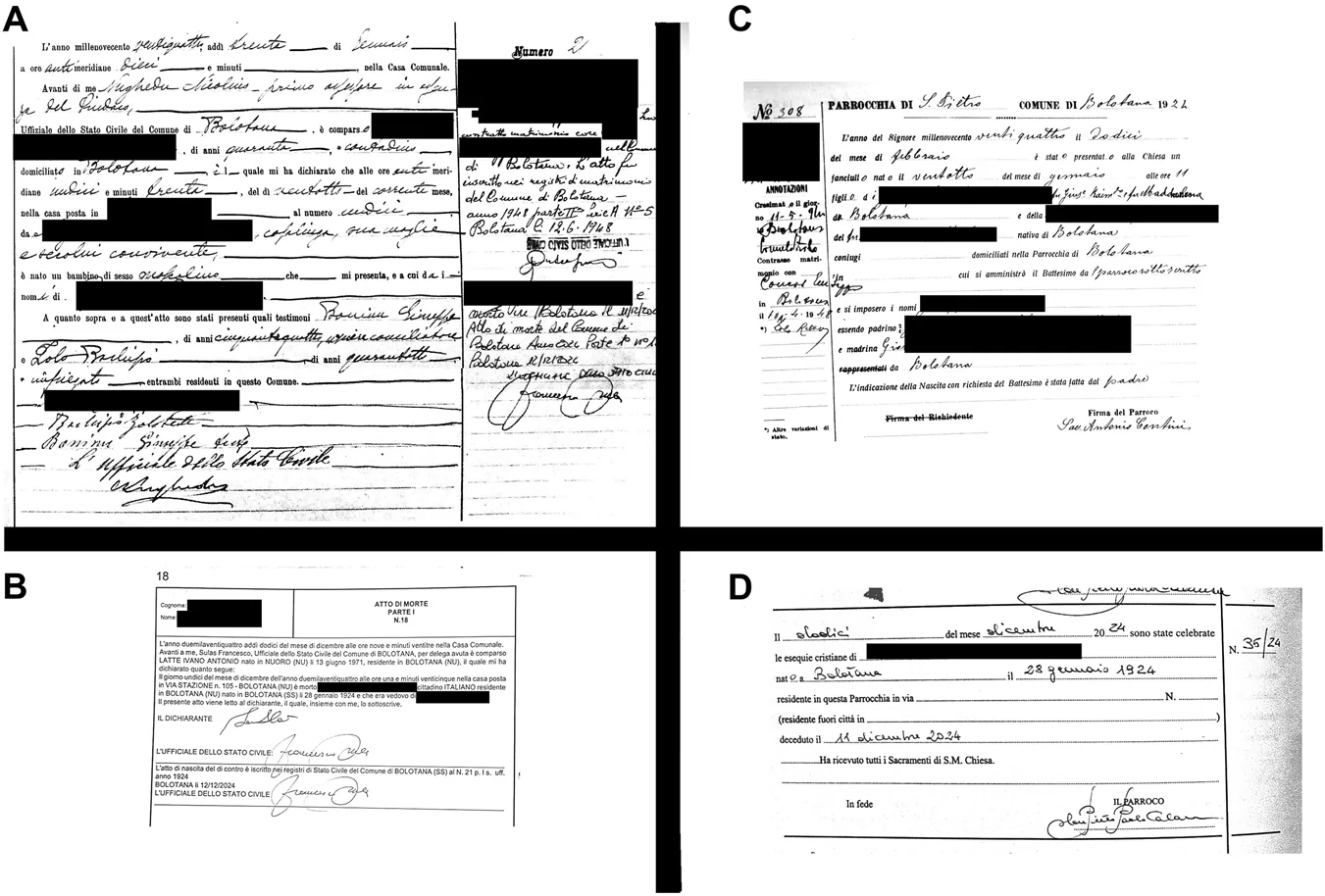
Cross-validation of civil and parish records for a Bolotana centenarian. **(A)** Birth certificate from the Bolotana municipal civil registry; **(B)** death certificate from the municipal civil registry; **(C)** baptismal certificate from the parish archive; and **(D)** ecclesiastical death certificate from the parish archive. The independent civil and church records contain concordant demographic and chronological information, supporting linkage to the same individual and validation of age at death. All personally identifying information has been redacted to protect individual privacy. (Images reproduced with permission from the Civil Registry of the Municipality of Bolotana and the Parish Archive of Bolotana).

Concordance of names, dates, parentage, and other identifying information was used to confirm record linkage and validate age at death. A deidentified case-level age-validation dataset specifying the documentary sources used and their concordance is provided in the Supplementary Material.

### 2.2 Study populations and demographic data sources

The primary analysis characterized late-life survival, defined here as survival from age 70 onward and assessed using remaining life expectancy at age 70 (e_70_) and conditional survival from age 70 to exact age 100 [S_(70→100)_], in Bolotana during the 5-year period 2021–2025. Three nested contemporary populations—Nuoro Province, Sardinia, and Italy—were used as calendar-matched reference populations. In addition, pooled estimates for the 14 municipalities constituting the original Sardinian Blue Zone during 2015–2019 (Olshansky et al., 2026) and estimates for a composite of high-longevity Human Mortality Database populations during the same period were included as external longevity benchmarks. Because these latter estimates refer to an earlier calendar period, they were not treated as contemporaneous comparisons; the corresponding periods are shown explicitly in Table 1. For the primary Bolotana analysis, the analytical inputs consisted of observed deaths and person-years of exposure by sex and single year of age, aggregated over 2021–2025.

**TABLE 1:** Primary estimates of remaining life expectancy at age 70 and conditional survival from age 70 to 100 in Bolotana, contemporary reference populations, and longevity benchmarks. Calendar periods are shown explicitly. For Bolotana, 95% confidence intervals were estimated using 20,000 parametric Poisson-bootstrap replications; uncertainty measures or annual ranges for comparison populations are defined in the table footnotes.

Population	Period	Life expectancy at age 70, y	Conditional survival from age 70 to 100, %
​	​	Men	
Bolotana	2021–2025	16.02 (14.55–17.79)^#^	4.92 (1.58–12.76)^#^
Nuoro province	2021–2025	15.27 (14.68–15.79)*	1.74 (0.75–2.25)*
Sardinia	2021–2025	15.49 (14.87–15.93)*	1.69 (0.92–2.51)*
Italy (national)	2021–2025	15.48 (14.95–16.04)*	1.41 (0.83–2.07)*
Original Sardinian blue zone	2015–2019	16.93 (16.40–17.47)^§^	5.24 (3.06–8.11)^§^
Composite (HMD)	2015–2019	15.6^†^	2.5^†^
​	​	Women	
Bolotana	2021–2025	20.85 (19.42–22.43)^#^	13.83 (7.33–23.75)^#^
Nuoro province	2021–2025	18.61 (17.85–19.02)*	4.33 (3.32–5.39)*
Sardinia	2021–2025	18.40 (17.74–18.76)*	4.43 (2.65–5.66)*
Italy (national)	2021–2025	18.06 (17.64–18.49)*	3.54 (2.51–4.44)*
Original Sardinian blue zone	2015–2019	20.30 (19.84–20.75)^§^	8.76 (6.26–11.73)^§^
Composite (HMD)	2015–2019	18.8^†^	5.0^†^

^#^ Bolotana estimates were calculated from deaths and person-years of exposure aggregated over 2021–2025; uncertainty is expressed using parametric Poisson-bootstrap 95% confidence intervals.

^§^ Values for the original Sardinian Blue Zone are pooled life-table estimates for its 14 municipalities during 2015–2019 and were reproduced from Olshansky et al. (2026). These values are included as a historical longevity benchmark. A period-matched 2015–2019 sensitivity analysis for Bolotana is presented in Supplementary Table S2.

* Reference-population estimates are the arithmetic means of the official annual ISTAT, life-expectancy values for 2021–2025, with the minimum and maximum annual estimates shown in parentheses. The annual ranges describe temporal variation and should not be interpreted as confidence intervals.

^†^ Estimated values from Olshansky et al. (2026) for a composite of highest-longevity HMD, populations during 2015–2019 (Australia, Canada, France, Japan, Hong Kong, Switzerland) included as an external longevity benchmark rather than as a contemporaneous comparator.

Annual complete life tables for Nuoro Province, Sardinia, and Italy were obtained from the Italian National Institute of Statistics (ISTAT) (https://demo.istat.it/). For each population and sex, annual remaining life expectancy at age 70 (e_70_) was drawn from official life tables, and annual conditional survival from age 70 to exact age 100 [S_(70→100)_] was calculated from the corresponding life-table survivor functions as l_100_/l_70_. The five annual estimates were summarized by their arithmetic mean and minimum–maximum range. These values were used as descriptive contemporary benchmarks and were not subjected to inferential testing.

### 2.3 Primary period life-table construction for Bolotana

For the primary 2021–2025 analysis, sex-specific period life tables beginning at age 70 were constructed for Bolotana using deaths and person-years of exposure aggregated over 2021–2025, following standard demographic life-table procedures (Chiang, 1984; Preston et al., 2001). Closed single-year age intervals were used from ages 70 through 99, followed by an open interval at age 100 years and older. Central mortality rates were converted into annual conditional probabilities of death using the standard actuarial assumption that deaths were distributed uniformly within each closed age interval. The open interval at age 100 years and older was treated as terminal. Two indicators were derived from each sex-specific life table: remaining life expectancy at age 70,

$$e_{70}=\frac{T_{70}}{l_{70}}$$

and conditional survival from age 70 to exact age 100,

$$S_{\left(70\to 100\right)}=\frac{l_{100}}{l_{70}}$$

The complete mathematical derivation of the primary partial life tables, including the calculation of mortality probabilities, survivorship, person-years lived, total remaining person-years, and the treatment of the terminal age interval, is provided in Supplementary Appendix A.

### 2.4 Statistical uncertainty for the Bolotana estimates

Statistical uncertainty in the Bolotana estimates was quantified separately for men and women using a parametric Poisson bootstrap. This approach was selected because deaths are count outcomes observed over fixed person-years of exposure and because simulation-based methods are suitable for evaluating uncertainty in life-table indicators derived from small populations (Davison and Hinkley, 1997; Eayres and Williams, 2004; Scherbov and Ediev, 2011).

For each closed single-year age interval x=70,…,99 , the observed number of deaths was assumed to follow a Poisson distribution:

$$D_{x}∼\text{Poisson}\left(\mu_{x}\right)$$

where the fitted mean was

$${\hat{\mu }}_{x}={\hat{m}}_{x}E_{x}=D_{x}$$

In each bootstrap replication b , a new death count was generated independently at each age:

$$D_{x}^{\left(b\right)}∼\text{Poisson}\left(D_{x}\right),x=70,\ldots ,99,$$

while the observed person-years of exposure $E_{x}$ were held fixed. The simulated mortality rates were then calculated as

$$m_{x}^{\left(b\right)}=\frac{D_{x}^{\left(b\right)}}{E_{x}}$$

and a complete partial life table was reconstructed using the same procedures as in the primary analysis. Remaining life expectancy at age 70, $e_{70}^{\left(b\right)}$ , and conditional survival from age 70 to age 100, $S_{\left(70\to 100\right)}^{\left(b\right)}$ , were extracted from each simulated life table.

The mortality rate in the open interval at age 100 and older was held fixed at its observed value,

$$m_{100+}=\frac{D_{100+}}{E_{100+}}$$

in all bootstrap replications. Resampling the small number of deaths in this terminal interval could generate a zero-death realization, for which $m_{100+}=0$ and the open-interval quantity $L_{100+}=\frac{l_{100}}{m_{100+}}$ would be undefined. Fixing the terminal rate therefore ensured a finite and consistently defined closure of every simulated life table. Consequently, the resulting intervals quantify sampling variation in deaths at ages 70–99, conditional on the observed exposures and the estimated mortality rate at age 100 and older. The procedure was repeated 20,000 times for each sex. The 95% confidence limits were defined as the 2.5th and 97.5th percentiles of the resulting bootstrap distributions:

$${CI}_{95\%}\left(\theta \right)=\left[Q_{0.025}\left(\theta^{\left(b\right)}\right),Q_{0.975}\left(\theta^{\left(b\right)}\right)\right]$$

where θ represents either $e_{70}$ or $S_{70\to 100}$ . Percentile intervals are a standard form of bootstrap confidence interval (Davison and Hinkley, 1997).

Because simulated counts were generated from fitted Poisson distributions rather than by resampling individual observations, the procedure was parametric rather than non-parametric. All calculations were performed in R, and the complete analytical code and age-specific input data are provided in the Supplementary Material.

### 2.5 Sensitivity analyses

Two complementary sensitivity analyses were performed on the 2021–2025 Bolotana data to distinguish sensitivity to life-table construction from sensitivity to the stochastic model used to estimate uncertainty. Both analyses were conducted separately for men and women and were restricted to the Bolotana data.

First, sensitivity to the conversion of central mortality rates into annual probabilities of death was evaluated using a constant-force-of-mortality specification. In contrast to the primary actuarial conversion, the alternative probability of death for each closed single-year interval from ages 70 to 99 was calculated as

$$q_{x}^{CF}=1-\exp\left(-m_{x}\right)$$

with the corresponding survival probability

$$p_{x}^{CF}=\exp\left(-m_{x}\right)$$

Under this specification, person-years lived in each closed interval were calculated as

$$L_{x}^{CF}=\frac{l_{x}-l_{x+1}}{m_{x}}$$

when $m_{x}>0$ , and as $L_{x}^{CF}=l_{x}$ when $m_{x}=0$ . The 100+ interval was treated as in the primary analysis. Confidence intervals were estimated using the same 20,000-replication parametric Poisson bootstrap.

Second, sensitivity to the assumed distribution of age-specific deaths was evaluated using a binomial Monte Carlo procedure based on Chiang’s Bernoulli-trial framework and the simulation approach described by Andreev and Shkolnikov (Chiang, 1984; Andreev and Shkolnikov, 2010). For each closed age interval, the effective population at risk was derived from the observed deaths and the actuarial probability of death as

$${\tilde{N}}_{x}=\frac{D_{x}}{q_{x}}$$

which, under the primary assumption that deaths occur on average halfway through a single-year interval, is equivalent to

$${\tilde{N}}_{x}=E_{x}+0.5D_{x}$$

Age-specific death counts were then generated from binomial distributions with probabilities based on the observed actuarial $q_{x}$ values and the corresponding effective populations at risk. For each replication, the simulated probabilities of death were used to reconstruct the partial life table and calculate $e_{70}$ and S_(70→100)_. The 100+ interval was not assigned an independent binomial variation; its mortality rate was held fixed as in the primary Poisson-bootstrap analysis. This treatment is consistent with the open interval having $q_{100+}=1$ , while its number of entrants varies as a consequence of mortality simulated at younger ages. The procedure was repeated 20,000 times, and two-sided 95% confidence intervals were defined by the 2.5th and 97.5th percentiles of the simulated distributions.

The constant-force analysis produced alternative point estimates and Poisson-bootstrap intervals, whereas the binomial Monte Carlo analysis provided alternative uncertainty intervals for the primary actuarial life-table estimates. Results from the different procedures were compared to assess whether the substantive interpretation depended on either the life-table conversion assumption or the stochastic model for deaths. The sensitivity estimates were not combined or averaged. Results are reported in Table 2.

**TABLE 2:** Sensitivity of the Bolotana longevity estimates to alternative life-table and uncertainty specifications, 2021–2025.

Sex	Indicator	Primary actuarial estimate	95% CI (Poisson bootstrap)	95% binomial Monte Carlo interval	Constant-force estimate	95% constant-force Poisson-bootstrap CI
Men	e_70_	16.02	14.55–17.79	14.46–17.66	16.02	14.56–17.80
	S_(70→100)_	4.92	1.58–12.76	1.24–11.62	5.01	1.76–12.90
Women	e_70_	20.85	19.42–22.43	19.35–22.38	20.85	19.41–22.43
	S_(70→100)_	13.83	7.33–23.75	6.73–23.16	13.90	7.53–23.81

All estimates were calculated from the same Bolotana deaths and person-years of exposure for 2021–2025. The constant-force specification represents an alternative method of converting central mortality rates into annual death probabilities, whereas the binomial Monte Carlo procedure represents an alternative model for statistical uncertainty. The mortality rate in the open interval at age 100 years and older was held fixed during simulation. Survival estimates are expressed as percentages.

### 2.6 Temporal-window sensitivity analysis: Inclusion of 2020

To evaluate whether exclusion of the first year of the COVID-19 pandemic materially influenced the primary estimates, the complete primary analysis was repeated after extending the initial observation year from 2021 to 2020, leaving 2025 constant. Sex-specific deaths and person-years of exposure were reaggregated over the 6-year period, and e_70_ and S_(70→100)_ were recalculated using the same single-year actuarial life-table procedures and 20,000-replication parametric Poisson bootstrap used in the primary analysis. The 100+ mortality rate was again held fixed during bootstrap simulation. Because the 2020–2025 and 2021–2025 periods overlap substantially, this analysis was treated as a temporal-window sensitivity analysis rather than as an independent replication (Supplementary Table S1).

### 2.7 Period-matched sensitivity analysis, 2015–2019

To address the difference in calendar period between the primary Bolotana analysis (2021–2025) and the published estimates for the original Sardinian Blue Zone (2015–2019), an additional period-matched sensitivity analysis was conducted for Bolotana for 2015–2019. Age- and sex-specific resident population counts on January 1^st^ of each year from 2015 through 2020 were combined with individually validated deaths occurring among Bolotana residents during 2015–2019. Exact dates of births and deaths were used to allocate deaths within the corresponding Lexis triangles and to reconstruct person-years of exposure at each single-year-of-age.

For age *x* in calendar year *t*, exposure was reconstructed from consecutive January 1^st^ population stocks as

$$E_{x,t}=\frac{P_{x,t}+P_{x,t+1}}{2}+\frac{D_{x,t}^{L}+D_{x,t}^{U}}{6}$$

where $P_{x,t}$ and $P_{x,t+1}$ denote the resident population aged *x* at the beginning of consecutive calendar years, and $D_{x,t\;}^{L}$ and $D_{x,t}^{U}$ denote deaths occurring in the lower and upper Lexis triangles, respectively. Annual exposures were summed over 2015–2019. Person-time at age 100 years and older was calculated directly from the exact dates of attaining age 100, death, and the boundaries of the observation period.

At the oldest single ages, person-time was sparse, and in some cells the standard single-year actuarial formulation yielded undefined or unstable mortality estimates. Rather than apply continuity corrections, deaths and exposures were aggregated into 5-year age groups (70–74, 75–79, 80–84, 85–89, 90–94, and 95–99 years), followed by an open interval at age 100 years and older. Mortality within each closed age group was modeled under a constant-force assumption. For an interval *j* of width $n_{j}=5$ years, the central mortality rate was calculated as

$$m_{j}=\frac{D_{j}}{E_{j}}$$

with survival across the interval given by

$$p_{j}=\exp\left(-n_{j}m_{j}\right)$$

Person-years lived within each interval were calculated as

$$L_{j}=\frac{l_{j}-l_{j+n_{j}}}{m_{j}}$$

when $m_{j}>0$ , and as $L_{j}=n_{j}l_{j}$ when $m_{j}=0$ . The age group 100 years and older was treated as the terminal open interval. Remaining life expectancy at age 70 and conditional survival from age 70 to exact age 100 were then derived from the resulting grouped life table.

Statistical uncertainty was quantified using 20,000 parametric Poisson-bootstrap replications. Death counts in the six closed age groups were generated independently from Poisson distributions with means equal to the observed grouped death counts, while reconstructed exposures were held fixed. As in the primary analysis, the observed mortality rate in the 100+ interval was held fixed. Two-sided 95% confidence intervals were defined by the 2.5th and 97.5th percentiles of the bootstrap distributions.

For the period-matched reference comparison, estimates for Nuoro Province, Sardinia, and Italy were derived from official annual ISTAT life tables for 2015–2019. At each exact age, the annual life-table survivor and death functions were combined across the five calendar years to obtain pooled 2015–2019 estimates e_70_ and S_(70→100)_. The minimum and maximum of the five annual estimates are additionally reported as measures of temporal variation and are not confidence intervals. Published pooled 2015–2019 estimates and reported 95% confidence intervals for the original Sardinian Blue Zone were reproduced from Olshansky et al. (Olshansky et al., 2026). Because Bolotana required grouped-age estimation whereas the reference populations were derived from official or published life tables, these comparisons were considered descriptive and were not interpreted as formal tests of between-population differences (Supplementary Table S2).

### 2.8 Cohort-based Extreme Longevity Index

To complement the period life-table analysis, we calculated the Extreme Longevity Index (ELI) for individuals born in Bolotana during 1900–1925 (Poulain et al., 2004). ELI is defined as the proportion of the original birth cohort documented to have survived to ages 100+. The denominator (B) of ELI is the total number of live births registered in Bolotana during these birth cohorts, whereas the numerator (C_100_) is the number of age-validated individuals from the same cohorts documented to have reached exact age 100, irrespective of whether they subsequently died in Bolotana or elsewhere. Individuals who were alive at age 100 or older at the end of ascertainment were also included. The ELI was defined as

$$ELI=\frac{C_{100}}{B}\times 100,000.$$

A two-sided exact 95% confidence interval was calculated using the Clopper–Pearson method and expressed on the same scale (Clopper and Pearson, 1934). The resulting estimate was compared descriptively with the corresponding ELI for Italy reported for comparable birth cohorts (Poulain and Herm, 2025). Because a small proportion of individuals who emigrated from Bolotana could not be followed to a documented date and place of death, the ELI may slightly underestimate survival to age 100.

## 3 Results

During 2021–2025, the mean resident population of Bolotana was 2,266. The primary life-table analysis accumulated 3,138 person-years of exposure at ages 70 years and older, comprising 1,218 person-years in men and 1,920 person-years in women. During the same period, 149 deaths at ages 70 years and older were recorded (69 in men and 80 in women). Nineteen residents reached age 100–5 men and 14 women—of whom six were alive at the end of follow-up.

Sex-specific period life tables for ages 70 years and older were used to estimate remaining life expectancy at age 70, e_70_, and conditional survival from age 70 to exact age 100, S_(70→100)_. Table 1 presents the primary 2021–2025 estimates for Bolotana, with 95% Poisson-bootstrap confidence intervals, together with contemporary reference values for Nuoro Province, Sardinia, and Italy and the 2015–2019 historical longevity benchmarks. Methodological sensitivity analyses of the primary Bolotana estimates are summarized in Table 2. Temporal-window and period-matched sensitivity analyses are reported in Supplementary Tables S1, S2, respectively.

Among men, remaining life expectancy at age 70 in Bolotana was modestly higher than the corresponding contemporary reference values, whereas conditional survival S_(70→100)_ showed a more pronounced difference (Table 1). e_70_ was 16.02 years in Bolotana (95% CI, 14.55–17.79) compared with the 2021–2025 mean annual values of 15.27 years in Nuoro Province, 15.49 years in Sardinia, and 15.48 years in Italy. The Bolotana point estimate was also higher than the maximum annual values observed in Nuoro Province (15.79 years) and Sardinia (15.93 years), and close to the Italian maximum of 16.04 years. Similarly, S_(70→100)_ was 4.92% in Bolotana (95% CI, 1.58%–12.76%), compared with mean annual values of 1.74% in Nuoro Province, 1.69% in Sardinia, and 1.41% in Italy, and exceeded the highest annual value observed in each reference population. The wide confidence interval around the Bolotana survival estimate reflects the limited numbers of deaths and person-years at the oldest ages. Because the reference values represent descriptive annual summaries rather than inferential estimates, these comparisons should not be interpreted as formal tests of statistical significance.

Among women, both remaining life expectancy at age 70 and conditional survival from age 70–100 S_(70→100)_ were substantially higher in Bolotana than in the contemporary reference populations (Table 1). e_70_ was 20.85 years in Bolotana (95% CI, 19.42–22.43), compared with mean annual values of 18.61 years in Nuoro Province, 18.40 years in Sardinia, and 18.06 years in Italy. Bolotana estimate also exceeded the highest annual value observed in each reference population. Similarly, S_(70→100)_ was 13.83% in Bolotana (95% CI, 7.33%–23.75%), more than three times the corresponding mean annual values for Nuoro Province (4.33%), Sardinia (4.43%), and Italy (3.54%), and exceeded the highest annual value recorded in each reference population. Notably, the lower confidence limits for both Bolotana indicators remained above the corresponding annual reference ranges. Nevertheless, because the reference values represent descriptive annual summaries rather than inferential estimates, these comparisons should not be interpreted as formal tests of between-population differences. Female late-life survival therefore emerged as the principal distinguishing feature of Bolotana’s contemporary longevity profile.

When the primary 2021–2025 Bolotana estimates were compared descriptively with the published pooled 2015–2019 estimates for the 14 municipalities constituting the original Sardinian Blue Zone, different relative positions were observed by sex. Among men, Bolotana had slightly lower point estimates for both e_70_ and S_(70→100)_ than the original Blue Zone: 16.02 versus 16.93 years and 4.92% versus 5.24%, respectively. Among women, however, Bolotana had higher point estimates: e_70_ was 20.85 versus 20.30 years, and S_(70→100)_ was 13.83% versus 8.76% (Table 1). Because the estimates refer to different calendar periods these contrasts should be regarded only as comparisons with a historical longevity benchmark and not as evidence that Bolotana had superior survival. A period-matched comparison for 2015–2019 was therefore examined separately in a sensitivity analysis (Supplementary Table S2).

In the period-matched 2015–2019 sensitivity analysis, Bolotana showed lower late-life survival estimates than the original Sardinian Blue Zone for both sexes (Supplementary Table S2). Among men, e_70_ was 13.87 years in Bolotana (95% CI, 12.46–15.56) compared with 16.93 years (16.40–17.47) in the original Blue Zone, while S_(70→100)_ was 0.77% (0.11%–4.07%) versus 5.24% (3.06%–8.11%), respectively. Among women, e_70_ was 18.30 years in Bolotana (17.01–19.75) compared with 20.30 years (19.84–20.75) in the original Blue Zone, and S_(70→100)_ was 1.46% (0.33%–5.63%) versus 8.76% (6.26%–11.73%). Thus, the period-matched analysis showed a less favorable late-life survival profile in Bolotana than in the original Sardinian Blue Zone. Because the Bolotana estimates required grouped-age constant-force estimation whereas the original Blue Zone estimates were derived from pooled life tables, these comparisons remain descriptive and should not be interpreted as formal tests of between-population differences.

The methodological sensitivity analyses produced results closely aligned with the primary actuarial estimates (Table 2). The binomial Monte Carlo intervals were very similar to the corresponding Poisson-bootstrap intervals with only minor differences in interval width, although uncertainty in survival to age 100 remained substantial, particularly among men. Under the constant-force-of-mortality specification, e_70_ was 16.02 years for men and 20.85 years for women, while S_(70→100)_ was 5.01% and 13.90%, respectively. Differences from the primary actuarial estimates were negligible for e_70_ and less than 0.10 percentage points for S_(70→100)_, indicating that the substantive findings were not materially affected by either the alternative within-interval mortality assumption or the alternative model for statistical uncertainty.

Extending the observation window to include 2020 produced modestly lower estimates for both sexes but did not alter the pronounced female advantage in late-life survival. Among men, e_70_ decreased from 16.02 to 15.58 years and S_(70→100)_ from 4.92% to 3.93%; among women, e_70_ decreased from 20.85 to 20.37 years and S_(70→100)_ from 13.83% to 12.48% (Supplementary Table S1).

As a complementary cohort-based analysis, 38 of the 3,060 individuals born in Bolotana during 1900–1925 were documented as having reached age 100. This corresponded to an Extreme Longevity Index of 1,242 centenarians per 100,000 live births (exact 95% CI, 880–1,701). Because the ELI reflects survival from birth and is influenced by early- and midlife mortality as well as mortality at advanced ages, it was treated as a complementary historical indicator rather than as a direct measure of contemporary late-life survival. The cohort-based findings therefore provide additional historical evidence of a relatively high frequency of survival to age 100 in Bolotana, while remaining conceptually distinct from the period life-table estimates.

## 4 Discussion

The present study identified an unusually favorable late-life survival profile in Bolotana during 2021–2025, with the clearest advantage observed among women. Female remaining life expectancy at age 70 was 20.85 years, and conditional survival from age 70 to age 100 was 13.83%; both exceeded the mean and the highest annual values observed in Nuoro Province, Sardinia, and Italy during the same period. Female estimates for Bolotana during the primary observation period were also exceptionally high relative to longevity benchmarks from the Human Mortality Database; among the populations examined by Olshansky et al. (Olshansky et al., 2026), Bolotana women ranked second only to Hong Kong for both e_70_ and S_(70→100)_. Overall, Bolotana women exhibited a late-life survival profile among the most favorable observed internationally, with S_(70→100)_ more than three times the corresponding value in U.S. women.

Among men, remaining life expectancy at age 70 was only modestly higher than the corresponding contemporary reference means, whereas survival to age 100 was higher than the annual ranges observed in all three reference populations. However, the wide confidence interval around the male survival estimate reflects the substantial statistical uncertainty inherent in estimating extreme-age survival in a population of this size. Because the reference values were descriptive annual summaries rather than inferential estimates, these comparisons should not be interpreted as formal tests of statistical significance.

Bolotana’s predominantly female longevity and survival advantage during 2021–2025 contrasts with that historically described in the original Sardinian Blue Zone, where exceptional longevity was characterized particularly by favorable male survival and a comparatively small sex difference (Poulain et al., 2004). This contrast suggest that the sex-specific expression of exceptional late-life survival in Sardinia may vary across communities and calendar periods rather than conform to a single demographic pattern. Such heterogeneity could reflect differences in cohort composition, environmental, social, behavioral, or other population-specific characteristics, although the present demographic data is not designed to distinguish among these possible explanations.

Comparison with the published 2015–2019 estimates for the original Sardinian Blue Zone provides useful historical context but cannot, by itself, establish the relative magnitude of Bolotana’s longevity advantage. In the primary 2021–2025 analysis, Bolotana showed slightly lower male point estimates than the original Blue Zone (Poulain et al., 2004) but higher female estimates, particularly for survival from age 70 to 100. However, because the two sets of estimates refer to different calendar periods, these contrasts cannot be interpreted as evidence of equivalence or superiority. A period-matched 2015–2019 sensitivity analysis was therefore undertaken to provide a more appropriate temporal comparison.

The methodological sensitivity analyses supported the robustness of the primary 2021–2025 estimates. Values obtained under the constant-force-of-mortality specification were nearly identical to the primary actuarial estimates, and the alternative binomial Monte Carlo procedure yielded a similar characterization of statistical uncertainty. Extending the observation window to include year 2020 modestly reduced the estimates for both sexes but preserved the pronounced female survival advantage. In contrast, the period-matched 2015–2019 sensitivity analysis yielded a less pronounced late-life survival profile, particularly for survival to age 100. Overall, these findings indicate that the results were robust to alternative life-table and uncertainty assumptions, but that the magnitude of Bolotana’s late-life survival advantage was sensitive to the calendar period examined.

The cohort-based analysis provided complementary historical evidence of survival to age 100 in Bolotana. Among individuals born in Bolotana during 1900–1925, the Extreme Longevity Index was 1,242 centenarians per 100,000 live births, compared with a reported Italian value of 793 per 100,000 for comparable birth cohorts (Poulain and Herm, 2025). Unlike the period life-table indicators, however, the ELI measures realized survival from birth to age 100 and is therefore influenced by mortality throughout the life course, including mortality at younger and middle ages Differences in historical mortality conditions across populations may therefore affect ELI comparisons independently of mortality at advanced ages. For this reason, the Bolotana–Italy comparison should be interpreted descriptively and cautiously. In addition, incomplete follow-up of a small proportion of emigrants may have resulted in slight under-ascertainment of individuals who reached age 100 outside Bolotana.

Several mechanisms could plausibly contribute to the pronounced female survival advantage observed during 2021–2025, including genetic background, dietary patterns, physical activity, family support, social cohesion, continuity of residence, and access to care. However, the present demographic analysis cannot determine whether any of these factors contributed to the observed pattern or assess their relative importance. Bolotana’s high residential continuity and the availability of independent civil and parish records strengthen confidence in the demographic ascertainment, but they do not explain the survival advantage. Comparative individual-level studies will be required to evaluate potential biological, behavioral, and social determinants of exceptional longevity and survival among females in Bolotana.

The study has several important limitations. Bolotana is a relatively small population, and mortality estimates at the oldest ages are based on limited numbers of deaths and person-years of exposure, resulting in wide confidence intervals and substantial stochastic variability, particularly for survival to age 100. The bootstrap intervals are conditional on the observed exposures and on the fixed mortality rate in the open interval at age 100 years and older. The primary observation period was relatively short and included years affected by the COVID-19 pandemic. Bolotana’s mountainous setting and relatively low population density may also have influenced pandemic exposure; however, the absence of individual-level data on SARS-CoV-2 infection, vaccination, mobility, and cause-specific mortality precludes testing this possibility. Although extending the analysis to include 2020 produced only modest changes, the period-matched 2015–2019 sensitivity analysis yielded a less pronounced late-life survival profile, indicating that estimates—especially S_(70→100)_—are sensitive to the calendar window examined. Because person-time at the oldest single ages was sparse in 2015–2019, that sensitivity analysis required aggregation into 5-year age groups and a constant-force mortality formulation, which limits exact methodological comparability with the primary analysis and with published reference life tables. Period life table estimates describe mortality conditions during the specified observation period and should not be interpreted as the lifetime experience of an actual birth cohort.

The ELI, conversely, reflects realized survival from birth to age 100 and is therefore influenced by mortality throughout the life course; historical differences in early- and midlife mortality limit direct comparison with other populations but do not themselves constitute bias in the ELI. Incomplete follow-up of a small proportion of emigrants may, however, have resulted in under-ascertainment of individuals who reached age 100 outside Bolotana and thus a slight downward bias in the estimated ELI. Finally, comparisons with the reference populations were descriptive and may be influenced by differences in population size, annual mortality variation, and to a lesser extent, life table construction.

Taken together, the age-validation evidence, primary period life table estimates, methodological and temporal sensitivity analyses, and cohort-based findings support consideration of Bolotana as a candidate longevity blue zone. The primary 2021–2025 analysis was distinguished particularly by a pronounced female survival advantage, although the period-matched sensitivity analysis indicates that the magnitude of this advantage is not invariant across short calendar windows. The present evidence therefore does not yet justify classifying Bolotana as an established longevity blue zone. Longer observation periods, continued demographic validation, and individual-level comparative studies will be required to determine the persistence and possible determinants of its exceptional longevity profile.

## Data Availability

The original contributions presented in the study are included in the article/Supplementary Material, further inquiries can be directed to the corresponding author.
