# Supplementary material for: A new blue zone? exceptional late-life survival in Bolotana, Sardinia
Source: Front Aging. 2026 Oct 5;7:1951042. doi: 10.3389/fragi.2026.1951042 (PMC13679626; doi:10.3389/fragi.2026.1951042)
Supplement: Supplementary file 1 [file Supplementaryfile1.docx]

**Appendix A.** Derivation of the Bolotana partial period life tables

Annual deaths and person-years of exposure were pooled across the five-year study period separately by sex, and single year of age. For each age *x* and sex *s*, the aggregated values were calculated as:

$$\boldsymbol{D}_{\boldsymbol{x}}^{\boldsymbol{s}}\boldsymbol{=}\sum_{\boldsymbol{y=2021}}^{\boldsymbol{2025}} \boldsymbol{D}_{\boldsymbol{x,y}}^{\boldsymbol{s}}$$

$$\boldsymbol{E}_{\boldsymbol{x}}^{\boldsymbol{s}}\boldsymbol{=}\sum_{\boldsymbol{y=2021}}^{\boldsymbol{2025}} \boldsymbol{E}_{\boldsymbol{x,y}}^{\boldsymbol{s}}$$

For each sex and age interval, the central mortality rate was calculated as:

$$m_{x}=\frac{D_{x}}{E_{x}}$$

where $D_{x}$is the observed number of deaths and $E_{x}$is the corresponding person-years of exposure. For the closed single-year intervals from ages 70 through 99, the probability of dying within the interval was calculated as

$$q_{x}=\frac{m_{x}}{1+0.5m_{x}}$$

assuming that deaths occurred, on average, halfway through each one-year interval. The corresponding probability of surviving the interval was

$$p_{x}=1-q_{x}$$

The life table was initialized with an arbitrary radix of $l_{70}=100,000$survivors. The number surviving to each subsequent exact age was calculated recursively as

$$l_{x+1}=l_{x}p_{x}$$

and the number of life-table deaths in each interval was

$$d_{x}=l_{x}q_{x}$$

For ages 70–99, person-years lived within each interval were calculated as

$$L_{x}=\frac{l_{x}+l_{x+1}}{2}$$

The age group 100 years and older was treated as the terminal open interval. Its mortality rate was calculated from the observed deaths and exposure as

$$m_{100+}=\frac{D_{100+}}{E_{100+}}$$

with $q_{100+}=1$. Person-years lived in the open interval were estimated as

$$L_{100+}=\frac{l_{100}}{m_{100+}}$$

Total person-years remaining above age $x$were obtained by summing $L_{x}$over all subsequent age intervals:

$$T_{x}=\sum_{j=x}^{100+} L_{j}$$

Remaining life expectancy at age 70 was then calculated as

$$e_{70}=\frac{T_{70}}{l_{70}}$$

and conditional survival from age 70 to exact age 100 was calculated as

$$S_{(70\to100)}=\frac{l_{100}}{l_{70}}$$
