# Supplementary material for: A new blue zone? exceptional late-life survival in Bolotana, Sardinia
Source: Front Aging. 2026 Oct 5;7:1951042. doi: 10.3389/fragi.2026.1951042 (PMC13679626; doi:10.3389/fragi.2026.1951042)
Supplement: Supplementary file 2 [file DataSheet1.zip › Data/Supplementary Table S1.docx]

Supplementary Table S1. Temporal-window sensitivity of late-life survival estimates in Bolotana to inclusion of the year 2020.

| Sex | Indicator | Primary analysis, 2021–2025 | Temporal sensitivity, 2020–2025 | Change |
| --- | --- | --- | --- | --- |
| Men | $e_{70}$, years | 16.02 (14.55–17.79) | 15.58 (14.22–17.18) | −0.44 years |
|  | $S_{(70\to100)}$, % | 4.92 (1.58–12.76) | 3.93 (1.24–10.25) | −0.99 percentage points |
| Women | $e_{70}$, years | 20.85 (19.42–22.43) | 20.37 (19.07–21.80) | −0.48 years |
|  | $S_{(70\to100)}$, % | 13.83 (7.33–23.75) | 12.48 (6.95–20.75) | −1.35 percentage points |

Note. Values are point estimates with 95% parametric Poisson-bootstrap confidence intervals in parentheses. The 2020–2025 sensitivity analysis used the same actuarial life-table procedure and 20,000-replication parametric Poisson bootstrap as the primary 2021–2025 analysis. Changes represent the 2020–2025 estimate minus the corresponding 2021–2025 estimate and are expressed in years for e_70_ and percentage points for S_(70→100)_.

.
