# Supplementary material for: A new blue zone? exceptional late-life survival in Bolotana, Sardinia
Source: Front Aging. 2026 Oct 5;7:1951042. doi: 10.3389/fragi.2026.1951042 (PMC13679626; doi:10.3389/fragi.2026.1951042)
Supplement: Supplementary file 2 [file DataSheet1.zip › Data/Supplementary Table S2.docx]

Supplementary Table S2. Period-matched sensitivity analysis of late-life survival in Bolotana and reference populations, 2015–2019.

| Population, 2015–2019 | Men $e_{70}$, years | Men $S_{(70\to100)}$, % | Women $e_{70}$, years | Women $S_{(70\to100)}$, % |
| --- | --- | --- | --- | --- |
| Bolotana | 13.87 (12.46–15.56)^#^ | 0.77 (0.11–4.07)^#^ | 18.30 (17.01–19.75)^#^ | 1.46 (0.33–5.63)^#^ |
| Original Sardinian blue zone | 16.93 (16.40–17.47)^§^ | 5.24 (3.06–8.11)^§^ | 20.30 (19.84–20.75)^§^ | 8.76 (6.26–11.73)^§^ |
| Nuoro Province | 15.40 [15.24–15.61]* | 2.42 [1.88–3.47]* | 18.67 [18.14–19.09]* | 5.12 [4.00–5.64]* |
| Sardinia | 15.50 [15.20–15.86]* | 1.80 [1.18–2.40]* | 18.46 [18.09–18.75]* | 4.46 [3.21–5.15]* |
| Italy (National) | 15.16 [14.82–15.54]* | 1.26 [0.93–1.56]* | 17.91 [17.59–18.26]* | 3.49 [2.71–4.06]* |

^#^ Bolotana estimates were derived from the 2015–2019 period-matched sensitivity analysis. Because person-time at the oldest single ages was sparse and did not support stable single-year actuarial estimates, deaths and reconstructed person-years of exposure were aggregated into five-year age groups (70–74 through 95–99 years) and analyzed using a constant-force mortality formulation, with age 100 years and older treated as the terminal open interval. Parentheses indicate 95% confidence intervals obtained from 20,000 parametric Poisson-bootstrap replications.

^§^ Values for the original Sardinian Blue Zone are pooled life-table estimates for its 14 municipalities during 2015–2019, reproduced from Olshansky et al. (2026); parentheses indicate the 95% confidence intervals reported in that study.

* Values for Nuoro Province, Sardinia, and Italy are pooled estimates derived from official annual life tables for 2015–2019. Square brackets indicate the minimum–maximum range of the five annual estimates and should not be interpreted as confidence intervals.

Because Bolotana required grouped-age estimation whereas the reference populations were derived from published or official life tables, comparisons are descriptive and should not be interpreted as formal tests of between-population differences.
