# Supplementary material for: A new blue zone? exceptional late-life survival in Bolotana, Sardinia
Source: Front Aging. 2026 Oct 5;7:1951042. doi: 10.3389/fragi.2026.1951042 (PMC13679626; doi:10.3389/fragi.2026.1951042)
Supplement: Supplementary file 2 [file DataSheet1.zip › Code/R2_code_Bolotana_CI_Monte_Carlo.docx]

```
# ============================================================
# CHIANG-ANDREEV MONTE CARLO CONFIDENCE INTERVALS
# Bolotana, ages 70 to 100+
# ============================================================

# Load the data
file_path <- "bolotana_data_2020_2025.txt"

# Adjust to your path
data <- read.csv(file_path)

# Basic checks
stopifnot(
  data$Age[1] == 70,
  tail(data$Age, 1) == 100,
  all(diff(data$Age) == 1),
  all(data$Exposure_M > 0),
  all(data$Exposure_F > 0)
)

life_table_from_q <- function(qx, mx_open, age) {

  n_age <- length(age)

  if (length(qx) != n_age) {
    stop("qx and age must have the same length.")
  }

  if (mx_open <= 0) {
    stop("The mortality rate in the open interval must be positive.")
  }

  # The final interval is open
  qx[n_age] <- 1

  # Survivorship, with l70 = 1
  lx <- numeric(n_age)
  lx[1] <- 1

  for (i in 2:n_age) {
    lx[i] <- lx[i - 1] * (1 - qx[i - 1])
  }

  # Person-years lived in each interval
  Lx <- numeric(n_age)

  # Closed one-year intervals: ages 70-99
  Lx[1:(n_age - 1)] <-
    (lx[1:(n_age - 1)] + lx[2:n_age]) / 2

  # Open interval: 100+
  Lx[n_age] <- lx[n_age] / mx_open

  # Total future person-years
  Tx <- rev(cumsum(rev(Lx)))

  # Remaining life expectancy
  ex <- Tx / lx

  list(
    age = age,
    qx = qx,
    lx = lx,
    Lx = Lx,
    Tx = Tx,
    ex = ex
  )
}

chiang_andreev_ci <- function(
    deaths,
    exposure,
    age,
    n_sim = 20000,
    seed = 12345
) {

  n_age <- length(age)

  if (length(deaths) != n_age ||
      length(exposure) != n_age) {
    stop("Deaths, exposure and age must have equal lengths.")
  }

  index_70  <- match(70, age)
  index_100 <- match(100, age)

  if (is.na(index_70) || is.na(index_100)) {
    stop("Ages 70 and 100 must be present.")
  }

  closed <- 1:(n_age - 1)

  # ----------------------------------------------------------
  # Observed life table
  # ----------------------------------------------------------

  mx <- deaths / exposure

  qx <- mx / (1 + 0.5 * mx)

  # Final interval is 100+
  qx[n_age] <- 1

  lt_observed <- life_table_from_q(
    qx = qx,
    mx_open = mx[n_age],
    age = age
  )

  point_e70 <- lt_observed$ex[index_70]

  point_S70_100 <-
    lt_observed$lx[index_100] /
    lt_observed$lx[index_70]

  # ----------------------------------------------------------
  # Estimated Bernoulli trial numbers
  #
  # N_tilde = D / q
  #
  # Given q = m/(1 + 0.5m), this is equivalent to:
  #
  # N_tilde = exposure + 0.5 * deaths
  # ----------------------------------------------------------

  n_trials <- floor(exposure + 0.5 * deaths)

  if (any(n_trials[closed] <= 0)) {
    stop("All closed intervals must have positive trial numbers.")
  }

  # floor() reproduces the behaviour of the original Excel
  # CRITBINOM function for non-integer numbers of trials.

  set.seed(seed)

  simulated_results <- matrix(
    NA_real_,
    nrow = n_sim,
    ncol = 2,
    dimnames = list(
      NULL,
      c("e70", "S70_100")
    )
  )

  for (b in seq_len(n_sim)) {

    # Simulated deaths in closed age intervals
    deaths_star <- rbinom(
      n = length(closed),
      size = n_trials[closed],
      prob = qx[closed]
    )

    # Simulated probabilities of death
    qx_star <- qx

    qx_star[closed] <-
      deaths_star / n_trials[closed]

    # Open interval remains q = 1
    qx_star[n_age] <- 1

    # Recalculate the life table
    lt_star <- life_table_from_q(
      qx = qx_star,
      mx_open = mx[n_age],
      age = age
    )

    simulated_results[b, "e70"] <-
      lt_star$ex[index_70]

    simulated_results[b, "S70_100"] <-
      lt_star$lx[index_100] /
      lt_star$lx[index_70]
  }

  # Rank-based empirical percentiles, corresponding closely
  # to the original Andreev-Shkolnikov description
  ci <- apply(
    simulated_results,
    2,
    quantile,
    probs = c(0.025, 0.975),
    type = 1,
    na.rm = TRUE
  )

  results <- data.frame(
    Indicator = c("e(70)", "S(70-100)"),
    Estimate = c(point_e70, point_S70_100),
    Lower_95 = c(
      ci["2.5%", "e70"],
      ci["2.5%", "S70_100"]
    ),
    Upper_95 = c(
      ci["97.5%", "e70"],
      ci["97.5%", "S70_100"]
    )
  )

  list(
    results = results,
    simulations = simulated_results,
    qx_observed = qx,
    n_trials = n_trials,
    life_table = lt_observed
  )
}

# Males
andreev_M <- chiang_andreev_ci(
  deaths = data$Deaths_M,
  exposure = data$Exposure_M,
  age = data$Age,
  n_sim = 20000,
  seed = 12345
)

# Females
andreev_F <- chiang_andreev_ci(
  deaths = data$Deaths_F,
  exposure = data$Exposure_F,
  age = data$Age,
  n_sim = 20000,
  seed = 54321
)

andreev_M$results
andreev_F$results
```
