# Supplementary material for: A new blue zone? exceptional late-life survival in Bolotana, Sardinia
Source: Front Aging. 2026 Oct 5;7:1951042. doi: 10.3389/fragi.2026.1951042 (PMC13679626; doi:10.3389/fragi.2026.1951042)
Supplement: Supplementary file 2 [file DataSheet1.zip › Code/R3_code_Bolotana_CI_CFM.docx]

```
life_table_cfm <- function(deaths, exposure, age) {

  if (length(deaths) != length(exposure) ||
      length(deaths) != length(age)) {
    stop("Deaths, exposure and age must have equal lengths.")
  }

  if (!all(diff(age) == 1)) {
    stop("Ages must be consecutive one-year ages.")
  }

  if (any(exposure <= 0)) {
    stop("All exposures must be positive.")
  }

  n_age <- length(age)
  closed <- 1:(n_age - 1)

  # Central mortality rates
  mx <- deaths / exposure

  if (mx[n_age] <= 0) {
    stop("Mortality in the final open interval must be positive.")
  }

  # Constant-force conversion
  qx <- 1 - exp(-mx)

  # Final interval is open
  qx[n_age] <- 1

  # Survivorship, with l70 = 1
  lx <- numeric(n_age)
  lx[1] <- 1

  for (i in 2:n_age) {
    lx[i] <- lx[i - 1] * exp(-mx[i - 1])
  }

  # Person-years lived
  Lx <- numeric(n_age)

  # Exact CFM calculation for closed intervals
  cfm_factor <- numeric(length(closed))

  zero_mortality <- mx[closed] == 0

  cfm_factor[zero_mortality] <- 1

  cfm_factor[!zero_mortality] <-
    -expm1(-mx[closed][!zero_mortality]) /
    mx[closed][!zero_mortality]

  Lx[closed] <- lx[closed] * cfm_factor

  # Final open interval
  Lx[n_age] <- lx[n_age] / mx[n_age]

  # Total person-years above each age
  Tx <- rev(cumsum(rev(Lx)))

  # Remaining life expectancy
  ex <- Tx / lx

  list(
    age = age,
    mx = mx,
    qx = qx,
    lx = lx,
    Lx = Lx,
    Tx = Tx,
    ex = ex
  )
}

lt_cfm_M <- life_table_cfm(
  deaths = data$Deaths_M,
  exposure = data$Exposure_M,
  age = data$Age
)

lt_cfm_F <- life_table_cfm(
  deaths = data$Deaths_F,
  exposure = data$Exposure_F,
  age = data$Age
)

index_70  <- match(70, data$Age)
index_100 <- match(100, data$Age)

cfm_e70_M <- lt_cfm_M$ex[index_70]
cfm_e70_F <- lt_cfm_F$ex[index_70]

cfm_S_M <-
  lt_cfm_M$lx[index_100] /
  lt_cfm_M$lx[index_70]

cfm_S_F <-
  lt_cfm_F$lx[index_100] /
  lt_cfm_F$lx[index_70]

cat("CFM e(70), males:", cfm_e70_M, "\n")
cat("CFM e(70), females:", cfm_e70_F, "\n")
cat("CFM S(70-100), males:", cfm_S_M, "\n")
cat("CFM S(70-100), females:", cfm_S_F, "\n")

bootstrap_cfm <- function(
    deaths,
    exposure,
    age,
    n_sim = 20000,
    seed = 12345
) {

  n_age <- length(age)
  closed <- 1:(n_age - 1)

  index_70  <- match(70, age)
  index_100 <- match(100, age)

  if (is.na(index_70) || is.na(index_100)) {
    stop("The data must include ages 70 and 100.")
  }

  # Observed CFM estimates
  lt_observed <- life_table_cfm(
    deaths = deaths,
    exposure = exposure,
    age = age
  )

  point_estimates <- c(
    e70 = lt_observed$ex[index_70],
    S70_100 =
      lt_observed$lx[index_100] /
      lt_observed$lx[index_70]
  )

  simulations <- matrix(
    NA_real_,
    nrow = n_sim,
    ncol = 2,
    dimnames = list(
      NULL,
      c("e70", "S70_100")
    )
  )

  set.seed(seed)

  for (b in seq_len(n_sim)) {

    deaths_star <- deaths

    # Simulate closed ages 70-99
    deaths_star[closed] <- rpois(
      n = length(closed),
      lambda = deaths[closed]
    )

    # Keep the observed 100+ mortality rate fixed
    deaths_star[n_age] <- deaths[n_age]

    lt_star <- life_table_cfm(
      deaths = deaths_star,
      exposure = exposure,
      age = age
    )

    simulations[b, "e70"] <-
      lt_star$ex[index_70]

    simulations[b, "S70_100"] <-
      lt_star$lx[index_100] /
      lt_star$lx[index_70]
  }

  ci <- apply(
    simulations,
    2,
    quantile,
    probs = c(0.025, 0.975),
    type = 1,
    na.rm = TRUE
  )

  results <- data.frame(
    Indicator = c("e(70)", "S(70-100)"),
    Estimate = c(
      point_estimates["e70"],
      point_estimates["S70_100"]
    ),
    Lower_95 = c(
      ci["2.5%", "e70"],
      ci["2.5%", "S70_100"]
    ),
    Upper_95 = c(
      ci["97.5%", "e70"],
      ci["97.5%", "S70_100"]
    )
  )

  list(
    results = results,
    simulations = simulations,
    life_table = lt_observed
  )
}

cfm_M <- bootstrap_cfm(
  deaths = data$Deaths_M,
  exposure = data$Exposure_M,
  age = data$Age,
  n_sim = 20000,
  seed = 12345
)

cfm_F <- bootstrap_cfm(
  deaths = data$Deaths_F,
  exposure = data$Exposure_F,
  age = data$Age,
  n_sim = 20000,
  seed = 54321
)

cfm_M$results
cfm_F$results
```
