# Supplementary material for: A new blue zone? exceptional late-life survival in Bolotana, Sardinia
Source: Front Aging. 2026 Oct 5;7:1951042. doi: 10.3389/fragi.2026.1951042 (PMC13679626; doi:10.3389/fragi.2026.1951042)
Supplement: Supplementary file 2 [file DataSheet1.zip › Code/R4_code_Bolotana_temporal_sensitivity.docx]

```
# Supplementary R code
# Temporal sensitivity analysis for Bolotana:
# primary period 2021-2025 versus extended period 2020-2025

primary_file  <- "C:/…/bolotana_data_2021_2025.txt"
extended_file <- "C:/…/bolotana_data_2020_2025.txt"

primary  <- read.csv(primary_file)
extended <- read.csv(extended_file)

check_data <- function(dat) {
  required <- c("Age", "Deaths_M", "Exposure_M", "Deaths_F", "Exposure_F")
  stopifnot(
    all(required %in% names(dat)),
    identical(dat$Age, 70:100),
    all(dat$Exposure_M > 0),
    all(dat$Exposure_F > 0)
  )
}

check_data(primary)
check_data(extended)

# Actuarial partial life table beginning at age 70.
# The row labelled 100 is treated as the open interval 100+.
actuarial_life_table <- function(deaths, exposure, age) {
  n <- length(age)
  mx <- deaths / exposure

  if (mx[n] <= 0) {
    stop("The mortality rate in the 100+ interval must be positive.")
  }

  # Closed one-year intervals, assuming a_x = 0.5
  qx <- mx / (1 + 0.5 * mx)
  qx[-n] <- pmin(qx[-n], 1)
  qx[n] <- 1

  # Survivorship, with l_70 = 1
  lx <- c(1, cumprod(1 - qx[-n]))

  # Person-years lived
  Lx <- numeric(n)
  Lx[-n] <- (lx[-n] + lx[-1]) / 2
  Lx[n] <- lx[n] / mx[n]

  Tx <- rev(cumsum(rev(Lx)))

  c(
    e70 = Tx[1],
    S70_100 = lx[match(100, age)]
  )
}

# Parametric Poisson bootstrap.
# Deaths at ages 70-99 are simulated; exposures and the observed 100+ rate
# are held fixed. The 95% CI is the empirical percentile interval.
poisson_bootstrap <- function(
    deaths, exposure, age,
    n_sim = 20000,
    seed = 12345
) {
  n <- length(age)
  observed <- actuarial_life_table(deaths, exposure, age)

  set.seed(seed)

  simulations <- replicate(n_sim, {
    deaths_star <- deaths
    deaths_star[-n] <- rpois(n - 1, lambda = deaths[-n])

    actuarial_life_table(deaths_star, exposure, age)
  })

  ci <- t(apply(
    simulations, 1, quantile,
    probs = c(0.025, 0.975),
    type = 7
  ))

  data.frame(
    Indicator = c("e(70)", "S(70-100)"),
    Estimate = unname(observed),
    Lower_95 = ci[, 1],
    Upper_95 = ci[, 2],
    row.names = NULL
  )
}

analyse_period <- function(dat, period) {
  male <- poisson_bootstrap(
    dat$Deaths_M, dat$Exposure_M, dat$Age,
    n_sim = 20000, seed = 12345
  )

  female <- poisson_bootstrap(
    dat$Deaths_F, dat$Exposure_F, dat$Age,
    n_sim = 20000, seed = 54321
  )

  out <- rbind(
    cbind(Period = period, Sex = "Male", male),
    cbind(Period = period, Sex = "Female", female)
  )

  # Express S(70-100) as a percentage
  survival <- out$Indicator == "S(70-100)"
  out[survival, c("Estimate", "Lower_95", "Upper_95")] <-
    100 * out[survival, c("Estimate", "Lower_95", "Upper_95")]

  out
}

results <- rbind(
  analyse_period(primary,  "2021-2025"),
  analyse_period(extended, "2020-2025")
)

results[c("Estimate", "Lower_95", "Upper_95")] <-
  lapply(results[c("Estimate", "Lower_95", "Upper_95")], round, 2)

print(results)
write.csv(results, "Bolotana_temporal_sensitivity_results.csv", row.names = FALSE)
```
