# Supplementary material for: A new blue zone? exceptional late-life survival in Bolotana, Sardinia
Source: Front Aging. 2026 Oct 5;7:1951042. doi: 10.3389/fragi.2026.1951042 (PMC13679626; doi:10.3389/fragi.2026.1951042)
Supplement: Supplementary file 2 [file DataSheet1.zip › Code/R5_code_Bolotana_grouped constant-force analysis_Poisson_bootstrap.docx]

```
# Bolotana 2015-2019 period-matched sensitivity analysis
# Five-year grouped constant-force life table + 20,000-replication
# parametric Poisson bootstrap.
#
# Closed groups: 70-74, 75-79, ..., 95-99.
# The 100+ mortality rate is held fixed in bootstrap replications,
# paralleling the treatment of the terminal interval in the primary analysis.

dat <- read.csv("bolotana_data_2015_2019_grouped.txt")

cf_grouped_life_table <- function(deaths, exposure, width, d100, e100) {
  stopifnot(
    length(deaths) == 6,
    length(exposure) == 6,
    length(width) == 6,
    all(exposure > 0),
    all(width > 0),
    d100 > 0,
    e100 > 0
  )

  mx <- deaths / exposure

  l <- 1
  L_total <- 0

  for (i in seq_along(mx)) {
    if (mx[i] > 0) {
      l_next <- l * exp(-mx[i] * width[i])
      L_i <- (l - l_next) / mx[i]
    } else {
      l_next <- l
      L_i <- l * width[i]
    }

    L_total <- L_total + L_i
    l <- l_next
  }

  S70_100 <- l

  # Open interval 100+
  m100 <- d100 / e100
  L100 <- l / m100

  e70 <- L_total + L100

  c(e70 = e70, S70_100 = S70_100)
}

poisson_bootstrap_grouped_cf <- function(
    deaths, exposure, width, d100, e100,
    n_sim = 20000, seed = 12345
) {
  observed <- cf_grouped_life_table(
    deaths, exposure, width, d100, e100
  )

  set.seed(seed)

  simulations <- replicate(n_sim, {
    deaths_star <- rpois(length(deaths), lambda = deaths)

    cf_grouped_life_table(
      deaths_star, exposure, width, d100, e100
    )
  })

  ci <- t(apply(
    simulations, 1, quantile,
    probs = c(0.025, 0.975),
    type = 7
  ))

  data.frame(
    Indicator = c("e(70)", "S(70-100)"),
    Estimate = c(observed["e70"], observed["S70_100"]),
    Lower_95 = ci[,1],
    Upper_95 = ci[,2],
    row.names = NULL
  )
}

closed <- dat[dat$Age_group != "100+", ]
terminal <- dat[dat$Age_group == "100+", ]

male <- poisson_bootstrap_grouped_cf(
  deaths = closed$Deaths_M,
  exposure = closed$Exposure_M,
  width = closed$Width,
  d100 = terminal$Deaths_M,
  e100 = terminal$Exposure_M,
  n_sim = 20000,
  seed = 12345
)

female <- poisson_bootstrap_grouped_cf(
  deaths = closed$Deaths_F,
  exposure = closed$Exposure_F,
  width = closed$Width,
  d100 = terminal$Deaths_F,
  e100 = terminal$Exposure_F,
  n_sim = 20000,
  seed = 54321
)

results <- rbind(
  cbind(Sex = "Male", male),
  cbind(Sex = "Female", female)
)

is_survival <- results$Indicator == "S(70-100)"
results[is_survival, c("Estimate","Lower_95","Upper_95")] <-
  100 * results[is_survival, c("Estimate","Lower_95","Upper_95")]

results[c("Estimate","Lower_95","Upper_95")] <-
  lapply(results[c("Estimate","Lower_95","Upper_95")], round, 2)

print(results)

write.csv(
  results,
  "Bolotana_2015_2019_grouped_CF_results.csv",
  row.names = FALSE
)
```
