# Supplementary material for: A new blue zone? exceptional late-life survival in Bolotana, Sardinia
Source: Front Aging. 2026 Oct 5;7:1951042. doi: 10.3389/fragi.2026.1951042 (PMC13679626; doi:10.3389/fragi.2026.1951042)
Supplement: Supplementary file 2 [file DataSheet1.zip › Code/R1_code_Bolotana_longevity_indicators.docx]

```
# Supplementary R code
# Primary actuarial life-table estimates and 95% parametric Poisson-bootstrap CIs
# Bolotana, 2021-2025

# Input file: the row labelled 100 must represent the open interval 100+
file_path <- "bolotana_data_2021_2025.txt"

# Adjust to your path
dat <- read.csv(file_path)

required <- c("Age", "Deaths_M", "Exposure_M", "Deaths_F", "Exposure_F")
stopifnot(
  all(required %in% names(dat)),
  all(dat$Age == 70:100),
  all(dat$Exposure_M > 0),
  all(dat$Exposure_F > 0)
)

# Actuarial partial life table beginning at age 70
actuarial_life_table <- function(deaths, exposure, age) {
  n <- length(age)

  stopifnot(
    length(deaths) == n,
    length(exposure) == n,
    all(diff(age) == 1),
    age[1] == 70,
    age[n] == 100,
    all(exposure > 0)
  )

  mx <- deaths / exposure
  if (mx[n] <= 0) stop("The mortality rate in the 100+ interval must be positive.")

  # Closed one-year intervals: actuarial conversion with a_x = 0.5
  qx <- mx / (1 + 0.5 * mx)
  qx[-n] <- pmin(qx[-n], 1)

  # Final open interval
  qx[n] <- 1

  # Survivorship, with l_70 = 1
  lx <- c(1, cumprod(1 - qx[-n]))

  # Person-years lived
  Lx <- numeric(n)
  Lx[-n] <- (lx[-n] + lx[-1]) / 2
  Lx[n] <- lx[n] / mx[n]

  Tx <- rev(cumsum(rev(Lx)))

  list(
    e70 = Tx[1] / lx[1],
    S70_100 = lx[match(100, age)] / lx[1],
    mx = mx,
    qx = qx,
    lx = lx,
    Lx = Lx,
    Tx = Tx
  )
}

# Parametric Poisson bootstrap
poisson_bootstrap <- function(
    deaths, exposure, age,
    n_sim = 20000,
    seed = 12345
) {
  n <- length(age)
  observed <- actuarial_life_table(deaths, exposure, age)

  set.seed(seed)

  simulations <- replicate(n_sim, {
    deaths_star <- deaths

    # Simulate deaths at ages 70-99; keep the observed 100+ rate fixed
    deaths_star[-n] <- rpois(n - 1, lambda = deaths[-n])

    lt_star <- actuarial_life_table(deaths_star, exposure, age)
    c(e70 = lt_star$e70, S70_100 = lt_star$S70_100)
  })

  ci <- t(apply(
    simulations, 1, quantile,
    probs = c(0.025, 0.975),
    type = 7
  ))

  data.frame(
    Indicator = c("e(70)", "S(70-100)"),
    Estimate = c(observed$e70, observed$S70_100),
    Lower_95 = ci[, 1],
    Upper_95 = ci[, 2],
    row.names = NULL
  )
}

male <- poisson_bootstrap(
  dat$Deaths_M, dat$Exposure_M, dat$Age,
  n_sim = 20000, seed = 12345
)

female <- poisson_bootstrap(
  dat$Deaths_F, dat$Exposure_F, dat$Age,
  n_sim = 20000, seed = 54321
)

results <- rbind(
  cbind(Sex = "Male", male),
  cbind(Sex = "Female", female)
)

# Convert survival estimates and limits from proportions to percentages
is_survival <- results$Indicator == "S(70-100)"
results[is_survival, c("Estimate", "Lower_95", "Upper_95")] <-
  100 * results[is_survival, c("Estimate", "Lower_95", "Upper_95")]

results[c("Estimate", "Lower_95", "Upper_95")] <-
  lapply(results[c("Estimate", "Lower_95", "Upper_95")], round, 2)

print(results)
write.csv(results, "Bolotana_primary_results.csv", row.names = FALSE)
```
